# Measuring cognitive flexibility: A brief review of neuropsychological, self-report, and neuroscientific approaches

**DOI:** 10.3389/fnhum.2024.1331960

**Published:** 2024-02-19

**Authors:** Kelly Hohl, Sanda Dolcos

**Affiliations:** ^1^Psychology Department, University of Illinois at Urbana-Champaign, Urbana, IL, United States; ^2^Beckman Institute for Advanced Science and Technology, University of Illinois at Urbana-Champaign, Urbana, IL, United States

**Keywords:** cognitive flexibility, resilience, assessment, interventions, brain-behavior

## Abstract

Cognitive flexibility involves dynamic processes that allow adaptation of our thinking and behavior in response to changing contextual demands. Despite a large consensus about its beneficial effects, cognitive flexibility is still poorly understood. In this mini review, we examined the main conceptualizations and approaches for assessing cognitive flexibility: (1) neuropsychological tasks, (2) self-report questionnaires, and (3) neuroscientific approaches. The reviewed evidence shows that the definition and assessment of cognitive flexibility are not unified within the field and suggests that a more consensual and consistent conceptualization and operationalization of this important concept is needed. We propose that an integrative behavior-brain-context approach can help advance our understanding of cognitive flexibility.

## Introduction

Adjustment to the ever-changing demands of everyday life has become essential for successfully navigating the constantly evolving and fast-paced world. At the core of this adjustment lies *cognitive flexibility*, which allows individuals to adapt their thinking and behavior in response to new circumstances and challenges. Cognitive flexibility (CF) has substantial benefits on multiple aspects of everyday life, from academic achievement to increased resilience and wellbeing (Diamond and Lee, 2011). For example, CF has been associated with overall academic achievement (Titz and Karbach, 2014), increased trait resilience against stress (Genet and Siemer, 2011), better quality of life (Davis et al., 2010), and increased mindfulness (Moore and Malinowski, 2009). Cognitive *in*flexibility, on the other hand, has been associated with ostensibly negative outcomes, such as an increased risk of developing depressive and anxiety disorders (Davis and Nolen-Hoeksema, 2000; Chamberlain et al., 2021). Additionally, inflexibility has been linked to personality traits, such as rigid decision-making styles characterizing schizophrenia and autism spectrum disorder (Tarasi et al., 2023), which researchers suggest might pave the way for maladaptive attitudes, such as conspiratorial thinking during the COVID-19 pandemic (Larsen et al., 2021).

Given the strong relationship between CF and important indicators of functioning, the last decade has seen an increased interest in the development of new assessments and interventions intended to improve CF. However, the success of this enterprise has been hindered by lack of a unified conceptualization and operationalization of CF within the field. Here, we start by outlining the various ways that CF is defined, provide our conceptualization, and describe the main types of approaches used to measure CF, including: (1) neuropsychological tasks, (2) self-report questionnaires, and (3) neuroscientific approaches. Then, we will introduce typical CF interventions. Finally, we will provide conclusions and highlight implications for future research.

## Defining cognitive flexibility

Cognitive flexibility is a difficult concept to define due to a variety of factors, including the interchangeable use of multiple concepts and a lack of unified conceptualizations or operational definitions. Historically, CF has been used interchangeably with psychological flexibility (Martin and Rubin, 1995; Lezak, 2004), coping flexibility (Cheng, 2001; Kato, 2012), mental flexibility (Oosterman et al., 2010; Matthew and Stemler, 2013), and explanatory flexibility (Fresco et al., 2007). In the neuroscience field, “cognitive flexibility” is often used interchangeably with “behavioral flexibility.” The “cognitive” part of CF refers to the mental ability to switch between cognitive sets or strategies in response to changing contextual demands (Scott, 1962; Ionescu, 2012); the “behavioral” part refers to the capacity to adaptively change behaviors in response to changing environmental demands (Ionescu, 2012; Brown and Tait, 2014; Uddin, 2021). The two constructs are closely intertwined, and in general, flexibility is believed to include both cognitive and behavioral components.

The lack of a unified account of CF has been highlighted in a comprehensive review of the literature which has identified four main conceptualizations of CF: *an ability or skill; a property of cognitive states*; *a personality trait*; and *an outcome of divergent thinking/measure for creativity* (Ionescu, 2012, 2017). The most common of these conceptualizations, which describes CF as an ability, considers flexibility as a core aspect of executive function (EF), and emphasizes the ability to shift between different tasks and goals as the central component of CF (Miyake et al., 2000). Shifting (the ability to shift attention from one criterion, rule, or task to another) is typically operationalized as “set-shifting” and “task switching,” with set-shifting being considered a more narrow, lower-level form of CF, and task-switching as the most complex form of CF (Bunge and Zelazo, 2006). Other definitions consider CF as involving multiple processes that encompass the coherent interaction of several other higher-order cognitive mechanisms, including EF (e.g., inhibition, working memory), salience detection, perception, coordination, monitoring, attention, or previous knowledge (Ionescu, 2012; Miyake and Friedman, 2012; Diamond, 2013). For instance, CF involves these interrelated processes to disengage from impulsivity or previous patterns (e.g., inhibition), consider alternatives (e.g., monitoring, updating), direct one’s focus toward pursuing new goals (e.g., attention, shifting), and achieve flexible behavior (Ionescu, 2012; Diamond, 2013; Dajani and Uddin, 2015; Morris and Mansell, 2018).

Approaches that view CF as a property of the cognitive system, rather than as an ability, consider flexibility as a property of the investigated processes (e.g., flexible language, flexible emotions), or as a property of the cognitive system as a whole (e.g., cognitive control) (Ionescu, 2012). The latter is seen as resulting from interactions among internal cognitive mechanisms and from the interactions of these internal mechanisms with external contextual factors (Ionescu, 2012, 2017). Approaches that view CF as a personality trait conceptualize flexibility as a part of “openness to experience,” and define it as awareness of multiple alternatives in any situation, the desire to be flexible, and self-efficacy in the capacity of being flexible [Martin and Rubin, 1995, cited by Chung et al. (2012)]. Finally, other definitions view CF as an essential ability for creativity, meaning the ability to change one’s perspective and to create something new (Dietrich and Kanso, 2010; Kleibeuker et al., 2013).

The conceptualizations described above suggest that CF is frequently used as an umbrella term to describe different psychological constructs. Ionescu (2017) recommends looking at these different constructs as sides or facets of CF, and to consider each of these sides as critical processes or mechanisms that contribute to overall CF (Ionescu, 2017). The idea that CF may emerge from the complex interactions of different cognitive mechanisms is supported by neuroscience research showing that flexible processing is the result of coordinated functioning among different neural networks (e.g., frontoparietal, frontostriatal) supporting different cognitive mechanisms - shifting/switching, inhibition, working memory, attention, and salience detection (Dajani and Uddin, 2015; Uddin et al., 2019; Uddin, 2021). Neuroscientific evidence can help further clarify the mechanisms that support CF, which would allow the development of a more precise conceptualization and operationalization of this important concept. In line with the views presented above, we define CF as a *property that emerges from optimal interactions among several cognitive and neural mechanisms to enable flexible adjustment of thoughts and behaviors to changing environmental demands*.

## Assessment approaches

The diverse conceptualizations of CF are also reflected in the heterogenous operationalizations and measurements of this concept across different domains. One type of commonly used measure consists of *neuropsychological tasks* that assess changes or switches in cognition and/or behavior in response to changing environmental contingencies. Another type of measure consists of *self-report questionnaires* that assess “changes in thoughts and behaviors” (Martin and Rubin, 1995) or “the ability to generate alternatives” (Dennis and Vander Wal, 2010). The fact that both neuropsychological tasks and self-report questionnaires are tests of CF suggests a unified underlying construct (Howlett et al., 2023). However, recent evidence shows little to no associations between them (Johnco et al., 2014); moreover, systematic meta-analyses from healthy and clinical populations suggest that neuropsychological tasks and self-report measures might not even assess the same construct (Howlett et al., 2021, 2023). This lack of relationship might be caused by the so-called task-impurity problem, which refers to the notion that these assessments might not capture exclusively just CF, and that successful completion of the CF measures requires both executive and non-executive functions and other capacities beyond just CF (Howlett et al., 2023). Importantly, these extra capacities seem to be different in the case of the two types of measures: self-report measures also require self-awareness, whereas neuropsychological tasks also require other aspects of EF and the involvement of distributed brain regions (Wildes et al., 2014). *Neuroscientific approaches* complement the information provided by neuropsychological tasks and self-report measures by identifying the brain mechanisms that underly CF. A growing body of studies using shifting and switching paradigms have identified the essential roles played by the lateral frontoparietal network (FPN) and the midcingulo-insular network (M-CIN) in supporting flexible behavior (Seeley et al., 2007; Sundermann and Pfleiderer, 2012). The FPN includes lateral prefrontal cortices (dorsolateral prefrontal cortex, dlPFC; ventrolateral prefrontal cortex, vlPFC; inferior frontal junction, IFJ), inferior parietal lobule, posterior inferior temporal lobes, and the M-CIN, which is also referred to as the salience network, and includes the bilateral anterior insula, anterior midcingulate cortex and subcortical nodes (i.e., amygdala and thalamus) (Rubia et al., 2006; Seeley et al., 2007).

Brief descriptions of these types of approaches, along with their advantages and disadvantages, are presented below.

## Neuropsychological tasks

Neuropsychological tasks are popular experimental techniques for assessing various EF processes and performance behaviors related to CF, such as shifting (or switching) mental sets, updating information in working memory, and inhibition of automatic responses (Miyake and Friedman, 2012; Friedman and Miyake, 2017). Common neuropsychological tasks include: (a) *task-switching paradigms*, where participants are instructed to perform two different tasks and must attend to the task as it changes demands (Monsell, 2003; Kiesel et al., 2010; Strobach et al., 2012), (b) *Dimensional Change Card Sort (DCCS) task*, where preschool-aged children are instructed to sort bivalent test cards according to one dimension (e.g., color) and then a second dimension (e.g., shape) (Zelazo, 2006), (c) *Trail Making Test (TMT)*, which includes two timed parts: (1) TMT-A, where participants draw lines to sequentially connect 25 numbers, and (2) TMT-B, where participants similarly draw a sequential line, but must alternate between numbers and letters (Reitan, 1958; Bowie and Harvey, 2006; Salthouse, 2011), (d) *Wisconsin Card Sorting Test (WCST)*, where participants sort response cards along certain dimensions (i.e., color, form or shape, and number) based on stimulus cards and changing rules with live feedback (Berg, 1948; Anderson et al., 1991; Rossell and David, 1997), (e) *Intra–Extradimensional (IED) Set-Shifting Task*, where participants are shown changing stimuli and must learn the rule and changing rules for stimuli categorization using corrective feedback (Sahakian and Owen, 1992; Cambridge Cognition, 2023), and (f) *Stroop Task or emotional Stroop Task*, where participants are instructed to name the color of a congruent or incongruent color-word stimulus (Stroop, 1935; MacLeod, 1992; Williams et al., 1996; Bugg et al., 2008).

Strengths of using neuropsychological measures include avoiding subjective biases (e.g., social desirability), implementing structured lab-based situations, and capturing automatic response processes (Dang et al., 2020; Howlett et al., 2021). As a challenge, neuropsychological tasks tend to have increased error variance compared to self-report measures, are more difficult to administer and score, and can be influenced by other aspects of cognition (e.g., task impurity problem) or practice effects (Dang et al., 2020; Howlett et al., 2021). Limitations in fully capturing aspects of real-world flexible behaviors that allow targeting of interventions has motivated an increased use of neuropsychological tasks in conjunction with self-report measures (Dennis and Vander Wal, 2010).

## Self-report questionnaires

Self-report questionnaires are commonly used measures for assessing cognitive and behavioral flexibility in everyday life, such as self-regulatory behaviors, flexible thinking patterns, shifting between situations and/or activities, perception of alternatives, and willingness and efficacy of flexibility. These measures are extensively used in intervention studies, typically reflecting the self-assessed application of CF abilities across a variety of situations and thought processes, which is relevant as generalizable intervention targets. Common self-report questionnaires for CF include: (a) *Cognitive Flexibility Inventory (CFI)*, where participants are asked to rate 20 items on whether they agree or disagree with statements corresponding to two subscales: “Alternatives” (i.e., the ability to perceive multiple alternative explanations for life occurrences and human behavior, and the ability to generate multiple alternative solutions to difficult situations) and “Control” (i.e., the tendency to perceive difficult situations as controllable) (Dennis and Vander Wal, 2010); (b) *Behavior Rating Inventory of Executive Function (BRIEF)*, where participants are asked to rate 75 items indicating whether a presented behavior has been a problem for them (e.g., “*I have trouble changing from one activity or task to another*”) over the past 6°months (Gioia et al., 1996, 2000, 2002; Roth et al., 2013); (c) *Cognitive Flexibility Scale (CFS)*, where participants are asked to rate 12 statements dealing with beliefs and feelings about flexible behaviors (e.g., “*I can find workable solutions to seemingly unsolvable problems*”) (Martin and Rubin, 1995); and (d) *Acceptance and Action Questionnaire (AAQ/AAQ-II)*, where participants are asked to rate seven items indicating one’s level of agreement to statements regarding psychological inflexibility or experiential avoidance (i.e., avoiding unpleasant thoughts/feelings) (e.g., “*My painful memories prevent me from having a fulfilling life*”) (Hayes et al., 2004; Bond et al., 2011).

Strengths of utilizing self-report measures include that they are typically quick, inexpensive, effective to administer, and often adapted across languages, cultures, and contexts. Importantly, self-report tools are often used clinically to assess one’s application of behaviors outside the laboratory setting, thus increasing ecological validity and generalizability (Dang et al., 2020). As a challenge, self-report measures are vulnerable to biases related to social desirability expectations and self-perceptive judgements (Dang et al., 2020; Howlett et al., 2021).

## Neuroscientific approaches

Studies investigating the neural substrates of CF employ non-invasive neuroimaging approaches in conjunction with neuropsychological and self-report measures. Common neuroscientific approaches used to measure CF include: *haemodynamic methods*, which depend on recordings of blood flow in the brain (e.g., functional magnetic resonance imaging/fMRI) (Reis and Judd, 2014; Ruff and Huettel, 2014; Carter and Shieh, 2015); *electrophysiological techniques*, which depend on recordings of electrical and/or magnetic fields in the brain (e.g., electroencephalography/EEG and event-related potentials/ERPs) (Kirschstein and Köhling, 2009; Kutas and Federmeier, 2011; Reis and Judd, 2014; Ruff and Huettel, 2014; Carter and Shieh, 2015); or *optical imaging methods*, which allow assessments of both haemodynamic-based and electrical signals using light transmission and reflection (e.g., event-related optical signals/EROS, near-infrared spectroscopy/NIRS) (Luker and Luker, 2008; Gratton and Fabiani, 2010).

Functional MRI, electrophysiological, and optical imaging studies using neuropsychological tasks have identified the core brain regions and networks supporting CF (Dajani and Uddin, 2015), along with task-dependent brain regions (Kim et al., 2011, 2012). Specifically, core CF brain regions common to these tasks include the ventrolateral and dorsolateral PFC, inferior frontal junction, anterior cingulate, right anterior insula, premotor cortex, inferior and superior parietal cortices, inferior temporal cortex, occipital cortex, and subcortical structures such as the caudate nucleus and thalamus (Kim et al., 2012; Niendam et al., 2012; Dajani and Uddin, 2015). Task-specific regions have also been identified. For instance, in neuroimaging studies, attention-shifting tasks have been shown to engage the dorsal portion of the premotor cortex and posterior PFC regions, whereas set-shifting tasks engage the frontopolar cortex (Kim et al., 2012), anterior PFC regions (in tasks with more dimensions, requiring more abstract switching) and mid-PFC regions (in tasks with fewer dimensions, requiring moderately abstract switching) (Kim et al., 2011). In an EEG study that incorporated a computerized cognitive training that improved switching performance, ERP analyses revealed an increase in fronto-central N2 (i.e., related to selecting appropriate responses to a target stimulus) amplitude; and the fronto-central correct response-locked negative component (Nc/CRN; related to perceived conflict even when a correct response is made) decreased in amplitude more after the flexibility training during task-switching blocks with neutral distractors, suggesting more conflict monitoring (Olfers and Band, 2018). In an optical imaging study, which involved preschool children using a personified object (i.e., doll) to prompt social interaction and EF development, results showed improvements in the DCCS task and increased activations in the left prefrontal regions when performing the DCCS, supporting the neurological development of EF in preschool children (Moriguchi et al., 2015).

Complementary evidence is provided by brain-behavior approaches that also incorporate self-report measures of CF. For example, a recent brain imaging study used a self-report measure of CF, the BRIEF scale (Gioia et al., 2000), to investigate behavioral and neural age-related differences in children and adults during the performance of a CF task (Kupis, 2021). Overall, the comprehensive knowledge provided by the combination of brain and behavioral studies has proven essential for advancing our understanding of the mechanisms underlying CF.

The use of neuroscientific methods in conjunction with neuropsychological tasks and self-report measures has the potential to improve our understanding of the mechanisms supporting CF. They also have important implications for targeted mental health interventions and for revealing responses that are unconscious or unable to be verbalized in a typical self-report (Kennedy et al., 2011; Bell et al., 2018). As a challenge, it is important to exercise caution when drawing conclusions from brain imaging studies. As mentioned earlier, CF assessments may be restricted by the task-impurity problem: successful completion of CF assessments requires both executive and non-executive functions, and therefore may engage other EF regions (Nowrangi et al., 2014), in addition to more specific CF regions.

## CF Interventions

Given the beneficial effects of CF on multiple aspects of everyday life, there is an increased focus on developing interventions to improve CF, especially in early and middle childhood, periods characterized by a high degree of plasticity and sensitivity to environmental input (Buttelmann and Karbach, 2017). Psychological research has suggested that CF can be conditioned and learned, often dependent on individual learning rates, frequency of changing contextual demands, or switching readiness (Braem and Egner, 2018; Wen et al., 2023). Similar to the definitions and measures, the current targets and structures of CF interventions are highly variable in the literature. CF intervention types have taken many forms, ranging from cognitive and physical exercise training to specialized curriculum programs (for reviews see Diamond, 2013; Karbach and Unger, 2014). The majority of the intervention studies train their participants to perform tasks that specifically target components relevant to CF - such as conducting multiple task-switching sessions to improve switching performance (Karbach and Kray, 2009). However, other studies take less direct training approaches, focusing on broad cognitive skill improvement to support the learning of CF, such as the effect of an active recess program with cognitive engagement on CF (measured as performance in the Trail Making Test: Ángel et al., 2021).

Training studies have shown that repeated practice is generally associated with benefits to executive functions (Diamond and Lee, 2011). However, such benefits are mostly limited to the specific aspect of the EF that is trained, reflecting near-transfer effectiveness (e.g., training on task-switching improves task-switching performance, and do not generalize to other cognitive or daily life skills) (Taatgen, 2013; Simons et al., 2016; Uddin, 2021). This limitation is especially relevant for certain training programs (especially “brain-training” programs) focused on preventing age-related decline, that have often been popularized under the guise of learning transfer while lacking quality scientific support (Simons et al., 2016). To address issues related to learning transfer and generalizability, an increasing number of recent studies have examined the contextual and individual factors that make people more or less flexible in different situations. For instance, studies have suggested that learning CF is strongly primed by environmental cues, such as frequent and forced/cued switching when learning a task, to essentially prompt faster adaptations to rule changes and independent flexible behavior to a novel task, therefore increasing flexibility and generalizability (Braem and Egner, 2018; Fröber et al., 2022; Wen et al., 2023). This new research has valuable implications for understanding the degree to which adjustments in CF are transferrable to new stimuli and tasks (reviewed in Egner and Siqi-Liu, 2024).

## General discussion

The aim of this mini-review was to summarize different conceptualizations, commonly used measures, and interventions targeting CF found across the scientific literature. Our review of the literature has identified multiple conceptualizations of CF in psychological research, reflecting two popular accounts of this construct: one as an ability, and the other as a property of various cognitive processes (Ionescu, 2012). To study either of these accounts, research frequently employs a single assessment method (i.e., neuropsychological tasks or self-report measures). However, more and more evidence is showing that these measures are only weakly associated or not even associated with each other, which suggests that they should not be used as proxies for each other (Howlett et al., 2021).

Our conceptualization of CF - as a property that emerges from optimal interactions among several cognitive and neural mechanisms to enable flexible adjustment of thoughts and behaviors to changing environmental demands - suggests that a more integrative framework anchored in behavior-brain-context interactions could provide a more unified account of CF. Based on this integrative perspective, multimethod approaches are recommended to both capture the multiple components of CF as a property of the cognitive system and to promote new insights regarding CF across multiple domains of functioning (Ionescu, 2012). Neuropsychological tasks, which measure responses “in the moment,” during structured situations, and are sensitive to within-personal experimental manipulations, should be complemented by self-report measures, which rely on the individual’s self-assessed CF across everyday behaviors and cognitions in a variety of contexts, and are more sensitive to capturing real-life behaviors. Neuroscientific evidence (e.g., fMRI) collected in tandem with neuropsychological tasks can help reveal the mechanisms supporting cognitive and behavioral flexibility. These behavior-brain approaches, complemented by knowledge of contextual and individual factors that make people more or less flexible in different situations, are especially relevant in intervention research. Such insights have the potential to identify interventions targets with a high likelihood of success. Additionally, multimethod findings can support initial steps for improving experimental procedures and intervention regimens to promote wellbeing, along with further refining the conceptualization of CF. Future research should continue using methods that assess multiple domains of functioning to assist with fully understanding CF as a unified psychological construct.

## Conclusions

The current world has been going through extended periods of instability and insecurity, including COVID-19, geopolitical events, and financial instability. The general perception of experiencing a permanent crisis is so pervasive that the term “permacrisis” was selected as the word of the year for 2022.^1^ To adapt to the ever-changing personal and institutional demands, it is essential to develop optimal levels of cognitive and behavioral flexibility, along with more adequate measures to assess CF. This can be accomplished by adopting a comprehensive assessment of CF that combines neuropsychological tasks, self-report questionnaires, and/or neuroscientific approaches, and by studying CF in the various contexts in which it appears.

## Author contributions

KH: Conceptualization, Investigation, Writing – original draft. SD: Writing – review & editing, Conceptualization, Investigation.

## Footnotes

1
https://theconversation.com/permacrisis-what-it-means-and-whyits-word-of-the-year-for-2022-194306
